# Multimodal monitoring of neutrophil activity during cardiac surgery

**DOI:** 10.3389/fimmu.2025.1504944

**Published:** 2025-03-13

**Authors:** Darko Jovanovski, Lisa Wohlgemuth, Pascal Max Lucien Lessing, Dominik Hüsken, Alexander Sebastian Koller, Bertram Dietrich Thomaß, Paul Müller, Marco Mannes, Sandra Nungeß, Marta Jovanovska, Bernd Mühling, Andreas Liebold, Markus Huber-Lang, David Alexander Christian Messerer

**Affiliations:** ^1^ Department of Cardiothoracic and Vascular Surgery, University Hospital Ulm, Ulm, Germany; ^2^ Institute of Clinical and Experimental Trauma Immunology, University Hospital Ulm, Ulm, Germany; ^3^ Institute of Transfusion Medicine, University Hospital Ulm, Ulm, Germany

**Keywords:** neutrophil granulocytes, platelets, cardiac surgery, ischemia-reperfusion injury, platelet-neutrophil complexes, platelet-activating factor, inflammation, thrombocytes

## Abstract

Cardiac surgery and the associated ischemia-reperfusion injury trigger an inflammatory response, which, in turn, can contribute to organ damage, prolonged hospitalization, and mortality. Therefore, the present study performed comprehensive monitoring of neutrophil-related inflammation in patients who underwent aortic valve surgery, including extracorporeal circulation. Neutrophil-related inflammation, as well as alterations in cellular physiology, phenotype, and function, were analyzed by flow cytometry, ELISA, and microscopy. Neutrophil activation occurred intraoperatively and preceded the upregulation of conventional inflammatory markers such as C-reactive protein and interleukin-6. Perioperatively, neutrophils maintained a stable response to platelet-activating factor (PAF) with regard to CD11b and CD66b expression but showed a decreased response in CD10. Postoperatively, neutrophils exhibited marked alterations in PAF-induced depolarization, while reactive oxygen species generation and phagocytic activity remained largely stable. Surprisingly, platelet-neutrophil complex formation was severely impaired intraoperatively but returned to normal levels postoperatively. Further studies are needed to elucidate the implications of these intraoperative and postoperative changes in neutrophil and platelet activity with respect to a potential immune dysfunction that temporarily increases susceptibility to infectious or hemostatic complications.

## 1 Introduction

Tissue injury and the related release of damage-associated molecular patterns (DAMPs) in conjunction with activation of the coagulation and immune systems can result in systemic inflammatory response syndrome (SIRS), for example, after severe physical injuries or during surgery (1–5). Cardiac surgery with the accompanying extracorporeal circulation results in surgical trauma and ischemia-reperfusion injury (IRI). Nonetheless, surgical interventions remain a cornerstone of the treatment of cardiac diseases, including vascular and valvular pathophysiologies (2, 6–8). However, despite medical progress, the 30-day lethality of cardiac surgical procedures remains at approximately 2–3% (8). Approximately one in three patients in a cardiac intensive care unit presents with SIRS, which, in turn, is associated with prolonged hospitalization, infectious complications such as sepsis, multiple organ dysfunction syndrome, and lethality (3, 6, 9).

Neutrophil granulocytes are the most abundant circulating immune cells, playing a pivotal role in SIRS, combating pathogens, and regeneration after injury (5, 9–12). Neutrophils can become activated by a plethora of stimuli such as microbe-associated molecular patterns (e.g., lipopolysaccharide, N-formylmethionine-leucyl-phenylalanine), complement cleavage products (e.g., C5a), interleukins (e.g., interleukin-8), and other substances, including the lipid-derived proinflammatory mediator platelet-activating factor (PAF) (5, 9, 12–15). Neutrophil activation results in a complex response pattern, which includes changes in cellular physiology such as a calcium influx, depolarization of the membrane potential (MP), and alkalization of the intracellular pH (pH_i_). Moreover, classical features of neutrophil activation are changes in cellular shape (13, 16–18) and degranulation of neutrophil granules releasing enzymes such as myeloperoxidase (11, 12). On a functional level, neutrophils respond with enhanced migratory activity, the release of neutrophil extracellular traps, an increase in phagocytic activity, and enhanced generation of reactive oxygen species (ROS) (9, 11, 12). Moreover, neutrophil activation involves crosstalk with other cells such as platelets, which, in turn, modulates neutrophil activity, results in the formation of platelet-neutrophil complexes (PNCs), and is part of a dangerous crosstalk between thrombosis and inflammation, frequently referred to as thromboinflammation (12, 13, 19).

Taken together, a better understanding of the neutrophil response before, during, and after cardiac surgery, its associated IRI, and inflammatory response, will contribute to the advancement of the pathophysiologic understanding, the generation of innovative monitoring and treatment approaches, and, ultimately, an improvement in patient care and survival. Therefore, the present study provides a multimodal neutrophil-centered immunomonitoring in patients with cardiac surgery requiring surgical aortic valve replacement accompanied by extracorporeal circulation, including markers of humoral inflammation as well as neutrophil physiology, phenotype, and function.

## 2 Material and methods

### 2.1 Patient recruitment

All experiments were performed in accordance with the Declaration of Helsinki, after ethical approval (number #452/21, Local Independent Ethics Committee of the University of Ulm) and obtaining written informed consent of the patients. The inclusion criteria were the ability to provide written informed consent, full legal age, and planned surgical aortic valve replacement with extracorporeal circulation. Exclusion criteria were revision procedures, active malignant disease with ongoing radio-/chemotherapy, and/or immunomodulatory medication. Blood was drawn on admission (A), which was usually the day before surgery, during the surgery (labeled ‘OR’, 45 min after the initiation of the extracorporeal circulation), and at 24, 48, and 120 h after the end of surgery (± 10%). Blood was drawn by peripheral venipuncture, through an existing peripheral venous catheter (A), from the arterial and the venous branch of the extracorporeal circulation (OR), from peripheral arterial catheters (if available, normally 24 h and 48 h after OR), or peripheral venous catheters (normally 120 h after OR). Samples from venous and arterial branches of the extracorporeal circulation showed no significant differences (data not shown), only data from the arterial branch is reported. Moreover, age- (± 10%) and sex-matched healthy volunteers (HVs) were included in the present study. HVs were recruited by announcements on public boards at Ulm University Hospital and study facilities. The inclusion criteria were no fever in the last seven days, no preexisting acute or malignant diseases, no immunomodulatory medication, no instable chronic preexisting diseases, the ability to provide written informed consent, and full legal age. The HVs were taking the following medications at the time of blood sampling: One HV was taking levothyroxine, one HV was taking pantoprazole and valsartan/hydrochlorothiazide, one HV was taking rivaroxaban and bisoprolol, and one HV was taking candesartan.

### 2.2 Hematological analysis and markers of humoral inflammation

Sodium, potassium, ionized calcium, and glucose were determined using a standard blood gas analyzer (ABL 800 Flex, Radiometer GmbH, Krefeld, Germany). Differential blood count (EDTA anticoagulated blood) and global coagulation parameters (activated partial thromboplastin time and the international normal ratio from citrate anticoagulated plasma) were determined using a standard hematology (Sysmex CN 2000, Sysmex, Kobe, Japan) and coagulation (BCS XP, Siemens, Marburg, Germany) analyzer, respectively, each according to the respective manufacturer’s standard protocol. C-reactive protein (CRP) was measured by a turbidimetric assay on a cobas c system (Roche, Penzberg, Germany). Procalcitonin (PCT), interleukin-6 (IL6), creatine phosphokinase-MB, and troponin-T were quantified by an electrochemiluminescence immunoassay on a cobas 8000/e 801 system (Roche). Urea, alanine transaminase, and creatine kinase were measured by a photometric assay on a Cobas c system (Roche) (all from lithium-heparin anticoagulated plasma). All the above-listed parameters were analyzed in cooperation with the Department of Clinical Chemistry of the University Hospital Ulm. For the analysis of myeloperoxidase (MPO) and matrix metalloproteinase 9 (MMP9), citrate anticoagulated whole blood was centrifuged for 10 min at 400 × g (adapted from (20)). The samples were stored at –80°C until further use. MPO was quantified using a LEGENDplex Human Vascular Inflammation Panel 1-S/P (#740809, Biolegend, San Diego, USA), while MMP9 was quantified by a standard enzyme-linked immunosorbent assay (#DY911, R&D Systems, Minneapolis, USA), in accordance to the instructions of the manufacturers.

### 2.3 Analysis of neutrophil phenotype and function

A total of 10 µL citrate anticoagulated blood was diluted with 5 µL phosphate-buffered saline (PBS) with calcium and magnesium (PBS^+/+^, #14040-091, Gibco, Thermo Fisher Scientific, Waltham, USA) and, if indicated in the figures, incubated with pharmacological inhibitors of 1 µM iloprost (#SML1651, Sigma-Aldrich, Steinheim, Germany), 1 mM ropivacaine (#ZYA1821/-22, Fresenius Kabi, Bad Homburg v. d. Höhe, Germany), or 2 µL anti-CD62P (#304904, Biolegend) or the respective buffer control for 10 min in a light-protected water bath at 37°C. Subsequently, the mixture was stimulated with either 1 µM PAF (#18779, Cayman Chemical Company, Ann Arbor, Michigan, USA) or buffer control in a total volume of 30 µL and incubated for 15 min at 37°C. The PAF concentration was chosen based on previous data to ensure maximal stimulation (13). Thereafter, for phenotype analysis, staining was performed with antibodies (all from BioLegend) against CD10 (PE-Cy7, 120 ng/mL, #312214; clone HI10a; isotype #400126), CD11b (APC, 600 ng/mL, #101212; clone M1/70; isotype #400612), and CD66b (APC-Cy7, 1 µg/mL, #305126; clone G10F5; no isotype available). For the analysis of the phagocytic activity, ROS generation, and PNC formation, 100 µL/mL fluorescent microspheres (Fluoresbrite BB Carboxylate 0.50 Micron Microspheres, Polysciences, Inc., Warrington, USA), 5 µM CellROX Deep Red (#C10422, Thermo Fisher Scientific), and an antibody against CD61 (PerCP, 2 µg/mL, #336410; clone VI-PL2; isotype #400148), respectively, were added as previously described (21). The samples were made up to a total volume of 50 µL by adding PBS^+/+^ and were incubated for 15 min in a light-protected water bath at 37°C. The stained and stimulated blood was transferred to 950 µL BD FACS Lysing Solution (#349202, BD Biosciences, San Jose, USA) and incubated for 30 min at room temperature in the dark. Following centrifugation at 340 × g for 5 min, the supernatant was discarded and the cells were resuspended in 100 µL PBS without calcium or magnesium (PBS**^−/−^**, #14190-094, Thermo Fisher Scientific) but with 1% bovine serum albumin (BSA, #A8022, Sigma Aldrich) Finally, the samples were maintained at 4°C in the dark until measurement.

### 2.4 Analysis of PNC formation

For flow cytometric analysis, PNCs were identified as CD61^+^-neutrophils as previously described (13, 21). For analysis by light microscopy (Axio Imager M1, Carl Zeiss Microscopy GmbH, Jena, Germany), 250 µl of citrate anticoagulated blood was diluted with 250 µL PBS^+/+^ and stimulated with either PBS^+/+^ as buffer control or 1 µM PAF. Blood smears were stained with the ‘Hemacolor Rapid staining of blood smear - staining set for microscopy’ (#111661, Merck, Darmstadt, Germany). For each sample, a minimum of 50 intact neutrophils per specimen were analyzed. Each neutrophil with at least one thrombocyte in direct proximity was considered to be a PNC (13, 21, 22).

### 2.5 Measurement of membrane potential, pH_i_, and cell shape

Granulocytes mainly consisting of neutrophil granulocytes (hereafter referred to as neutrophils) were purified by Ficoll separation, dextran sedimentation, and hypotonic lysis as previously described (14, 18). In brief, 9 mL of citrate anticoagulated blood was centrifuged for 10 min at 400 × g. After removing the plasma, the remaining blood cells were mixed with 0.9% sodium chloride (#1312813, Fresenius Kabi) up to a total volume of 20 mL, which was subsequently layered on ficoll (#17144003, Cytiva Sweden AB, Uppsala, Sweden) and centrifuged for 30 min (400 × g) followed by dextran sedimentation. Isolated granulocytes (purity usually > 95% as indicated by flow cytometry) were adjusted to a final concentration of 1 × 10^6^ cells/mL and resuspended in Hank’s balanced salt solution with calcium and magnesium (HBSS^+/+^, #14025050, Thermo Fisher Scientific, Darmstadt, Germany) containing 15 mM 2-(4-(2-hydroxyethyl)-1-piperazinyl)-ethanesulfonic acid (HEPES, #7365-45-9, Sigma). Cells were stained with 50 nM bis(1,3-dibutylbarbituric acid) trimethine oxonol (DiBAC_4_(3), #D8189, Merck, for measuring the MP) and 1 µM SNARF 5-(and-6)-carboxy-SNARF-1 (SNARF, #C1272, Invitrogen Thermo Fisher Scientific, Dreieich, Germany, for measuring pH_i_) in HBSS^+/+^ containing HEPES and maintained in a light-protected water bath at 37°C. After 20 min, the cells were centrifuged (5 min, 340 × g, room temperature) and resuspended in PBS^+/+^, followed by another incubation period of 10 min with 50 nM DiBAC_4_(3) before stimulation and measurement (14, 18). The granulocytes were stimulated with either 1 µM PAF or the respective buffer control as indicated. Cells were analyzed after 1, 5, and 10 min. The graphs report the cellular response at the time of the maximum for the respective parameter (1 min for MP, 5 min for pH_i_, 10 min for forward scatter (FSC)). Of note, the FSC is only a brief indicator for cellular size, because it more likely reflects a change in cellular shape as discussed before (13, 23).

### 2.6 Drug screening in vitro

A total of 10 µL citrate anticoagulated whole blood from healthy human volunteers (HVs, aged 21 – 23 years, otherwise inclusion criteria see above) was exposed to the drugs listed in **Supplementary Table 4** or PBS^+/+^ as buffer control for 10 min in a light-protected water bath at 37°C. The drug doses were determined by values reported in the literature and increased approximately 10- to 30-fold to briefly explore their effects on granulocytes and platelets. Subsequently, the samples were stained with anti-CD61 and anti-CD11b (as described above) and simultaneously exposed to 1 µM PAF for 30 min at 37°C in a light-protected water bath. The stained and stimulated blood was transferred to 950 µL BD FACS Lysing Solution for 30 min at room temperature in the dark. Following centrifugation at 340 × g for 5 min, the supernatant was discarded, the cells were resuspended in PBS^–/–^ containing 1% BSA, and the samples were maintained in the dark at 4°C until analysis by flow cytometry.

### 2.7 Platelet activation

To analyze platelet activation, 50 μL of citrate-anticoagulated blood was diluted with 562.5 µL HBSS^+/+^ as previously described (21). Subsequently, 10 µL of this diluted blood was added to 40 µL PBS^+/+^ including prior added stimuli (either buffer control or 1 µM PAF) and 1µL anti-CD61 and anti-CD62P (FITC, 8 µg/mL, #304904; isotype #400108, BioLegend). Following incubation for 10 min in a light-protected water bath at 37°C, 950 µL HBSS^++^ were added to the sample followed by immediate flow cytometry analysis. Platelets were identified by the properties of FSC, side scatter (SSC), and CD61 expression and were gated as described before (21).

### 2.8 Flow cytometric analysis

Doublets were removed by plotting the FSC area versus the height followed by analyzing linearity of both parameters. Neutrophils were identified based on their FSC and SSC area properties. The spillover between the fluorescence channels was corrected by a compensation matrix. For all antigens, appropriate isotype controls and single staining controls were performed (data not shown). For all experiments, a minimum of 3000 neutrophils or platelets were recorded using a BD FACSLyric (BD Biosciences) within a predefined stopping timer of 120 seconds.

### 2.9 Data analysis and statistics

**Figure 1:**
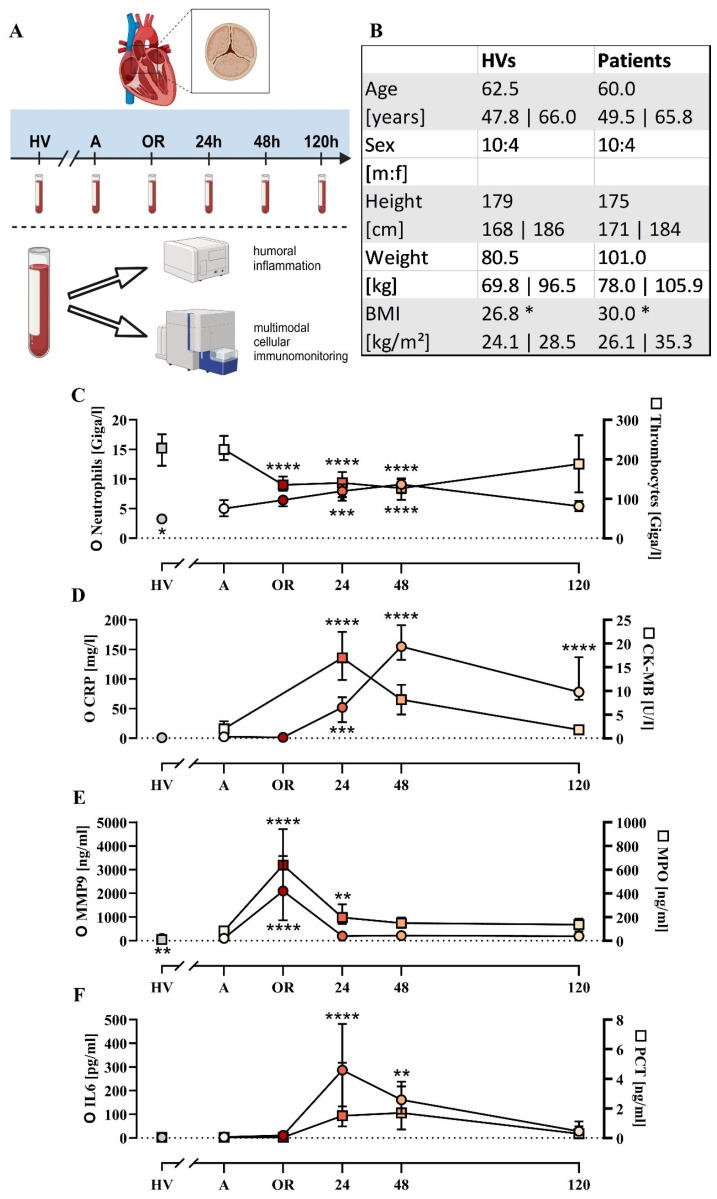
Cohort description and general parameters. **(A)** Summary of the experimental approach analyzing patients with cardiac surgery on admission (A), 45 min after the initiation of extracorporeal circulation in the operation room (OR), and 24, 48, and 120 h after the end of surgery in comparison to age- and sex-matched healthy volunteers (HVs). **(B)** General characteristics of the HVs and patients. Laboratory data for **(C)** neutrophil and thrombocyte count, **(D)** C-reactive protein (CRP) and creatine phosphokinase-MB (CK-MB), **(E)** matrix metalloproteinase 9 (MMP9) and myeloperoxidase (MPO), and **(F)** interleukin-6 (IL6) and procalcitonin (PCT). Median with interquartile range, n = 14. HVs vs. A: unpaired t test; A vs. OR, 24, 48, and 120 h post OR: ordinary one-way ANOVA in conjunction with a Dunnett’s multiple comparison test with *, **, ***, and **** indicating a p value of < 0.05, < 0.01, < 0.001, and <0.0001, respectively.

The flow cytometry data were analyzed using the custom-written, python-based flow cytometry analytics software ‘BFlow’ (BFlow Project, www.bflow.science, last accessed 05 April 2024). All data is presented as median with bars indicating the interquartile range, for example, median (25^th^ percentile | 75^th^ percentile), if not indicated otherwise. **Figure 1** was partially created with biorender.com. Data analysis was performed with licensed versions of Microsoft Excel 2019 (Microsoft, Redmond, USA) and GraphPad Prism 10 (GraphPad Software Inc., San Diego, USA). The study is intended to be an exploratory monocentric approach to monitor neutrophil function in perioperatively in patients with cardiac surgery. Therefore, several assumptions, including the justifications provided, were made. As most biological data are typically assumed to be normally distributed, and given that neither theoretical nor empirical evidence suggests otherwise, we performed a visual inspection of the present dataset to confirm this assumption. Consequently, a parametric approach was used for the statistical analysis. However, the resulting findings should be interpreted with these assumptions in mind, as they may overestimate the significance of the reported results. Furthermore, it could be argued that a correction for multiple testing would have been necessary; such a correction was not performed due to the exploratory nature of this study and the disparate nature of the parameters analyzed. A sample size calculation and effect size estimation were not performed because, to the authors’ knowledge, no information regarding the biological relevance of changes in the investigated parameters is currently available. For the statistical analysis comparing HVs with patients on admission, an unpaired t test was performed. The statistical description for patients during their course of stay was conducted by setting the admission time point as reference, the other time points were compared by applying an ordinary one-way analysis of variance (ANOVA). Further statistical testing was conducted as described in the figure legends. A p value < 0.05 was considered to be significant and marked with *, **, ***, and ****, indicating < 0.05, < 0.01, < 0.001, and <0.0001, respectively.

## 3 Results

### 3.1 Clinical characteristic and humoral inflammation

Fourteen patients were included as indicated in **Figure 1**, of whom 11/14 had preexisting arterial hypertension, 9/14 had hyperlipidemia, and 5/14 had preexisting type 2 diabetes mellitus. The duration of the operation including anesthesiology was 300 (270; 348) min and excluding anesthesiology (incision – suture) was 234 (173; 254) min. The duration of the extracorporeal circulation in total was 132 (123; 153) min and the ischemia time was 89 (77; 100) min. Access to the operation site was prepared by traditional sternotomy in 8/14 cases, by partial (upper) sternotomy in 3/14 cases, and by minimally invasive anterolateral thoracotomy in 3/14 cases. Replacement of the aortic valve was conducted by implanting biological valves in 9/14 cases and mechanical valves in 5/14 cases. An additional procedure was required in 7/14 patients, such as supracoronary ascending aortic replacement and/or bypass procedures. Apart from two patients requiring revision (one due to asymptomatic pericardial effusion treated by inferior pericardiotomy, one due to third-degree atrioventricular block treated by implantation of a pacemaker), no major complications were observed. The median stay in intensive care was 3 (2; 5) days postoperatively and the total length of the hospital stay was 9 (7; 11) days postoperatively. Of the 14 patients, 12 were discharged home, 1 was transferred to another hospital, and 1 was referred to a rehabilitation facility.

Basic monitoring of inflammation revealed largely unchanged leukocyte count, IL6, and PCT levels during surgery (**Figure 1**; **Supplementary Table 1**), but were increased postoperatively with peaks around the first or second postoperative day. By contrast, the MMP9 and MPO as markers of humoral inflammation were significantly elevated during surgery but returned to baseline 24 h post surgery (**Figure 1**). On a cellular level, erythrocytes and thrombocytes decreased slightly as expected during surgery (**Supplementary Tables 1**, **2**).

### 3.2 Changes in neutrophil phenotype, physiology, and function

**Figure 2:**
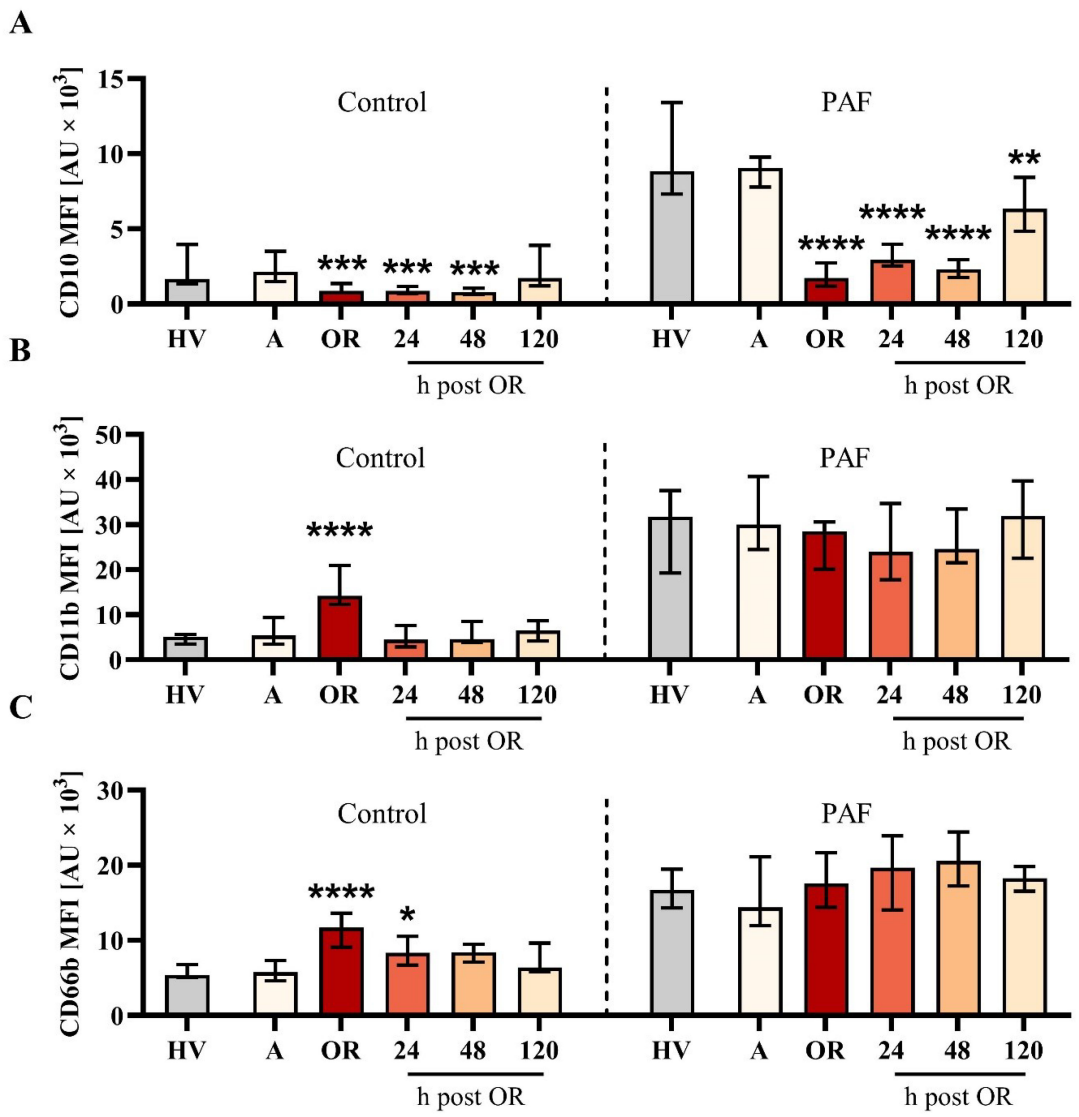
Analysis of neutrophil phenotype for **(A)** CD10, **(B)** CD11b, and **(C)** CD66b from patients with cardiac surgery on admission (A), 45 min after the initiation of extracorporeal circulation in the operation room (OR), and 24, 48, and 120 h after the end of surgery compared to healthy volunteers (HVs). Blood samples were stimulated with buffer control (control, left) or 1 µM platelet-activating factor (PAF, right). Median with interquartile range, n = 14. HVs vs. A: Unpaired t test; A vs. OR, 24, 48, and 120 h post OR: ordinary one-way ANOVA with *, **, ***, and **** indicating a p value of < 0.05, < 0.01, < 0.001, and <0.0001, respectively. MFI = median fluorescence intensity.

The elevation of the markers of humoral inflammation was clearly preceded by changes in the neutrophil phenotype (**Figure 2**). In detail, CD10 downregulation and CD11b and CD66b upregulation showed maximal changes intraoperatively. While CD10 remained decreased during the first two postoperative days, CD11b and, to a lesser extent, CD66b returned close to baseline levels one day postoperatively. Of note, neutrophil PAF-induced responsiveness with regard to CD10 but not to CD11b nor CD66b was impaired during the postoperative observation period. CD11b expression on neutrophils was significantly reduced by the addition of ropivacaine but not iloprost or an antibody against CD62P in neutrophils with or without additional exposure to PAF (**Table 1**). Of note, CD10 and CD11b largely did not correlate with conventional biomarkers postoperatively (**Supplementary Table 3**).

**Table 1:** Effect of iloprost, a blocking antibody against CD62P (anti-CD62P), and ropivacaine on neutrophil CD11b expression (upper panel) and platelet-neutrophil complex (PNC) formation as monitored by CD61 appearance on neutrophils (as an indicator of PNC formation, lower panel).

			HV	A	OR	24 h	48 h	120 h
CD11b	Ctrl	Iloprost	0.27	−0.13	0.01	−0.18	−0.37	−0.19
		anti-CD62P	0.51	0.31	0.05	0.65	0.36	0.53
		Ropivacaine	−0.25	−0.42**	−0.19	−0.49**	−0.63**	−0.54**
	PAF	Iloprost	−0.04	−0.09	0.01	−0.05	0.04	0.06
		anti-CD62P	0.05	0.08	0.02	0.03	0.04	0.10
		Ropivacaine	−0.54**	−0.59****	−0.36***	−0.57***	−0.58****	−0.49***
CD61	Ctrl	Iloprost	−0.83***	−0.56*	−0.28*	−0.80****	−0.71****	−0.65**
		anti-CD62P	−0.81***	−0.72***	−0.46**	−0.81****	−0.72****	−0.73***
		Ropivacaine	−0.60**	−0.40**	−0.37*	−0.57*	−0.62***	−0.59**
	PAF	Iloprost	−0.90****	−0.86****	−0.54*	−0.86****	−0.91****	−0.75****
		anti-CD62P	−0.82****	−0.78****	−0.72***	−0.89****	−0.87****	−0.74****
		Ropivacaine	−0.72****	−0.67****	−0.60**	−0.56****	−0.64****	−0.64****

Samples were analyzed from patients with cardiac surgery on admission (A), 45 min after the initiation of extracorporeal circulation in the operation room (OR), and 24, 48, and 120 h after the end of surgery compared to healthy volunteers (HV). At 10 min after exposure to the listed pharmacological modulator, the samples were additionally stimulated with buffer control (ctrl) or 1 µM platelet-activating factor (PAF). Data is normalized, 0.00 equals the respective sample without a pharmacological inhibitor except the corresponding buffer controls for stimulation with buffer control (Ctrl) or PAF. For CD11b, the median fluorescence intensity has been analyzed, for CD61, the percentage of PNCs is reported. Blue indicates a decrease, green indicates an increase. Median, n = 14, unpaired t test for the non-normalized data, with *, **, ***, and **** indicating a p value of <0.05, < 0.01, < 0.001, and <0.0001, respectively.

In an exploratory *in vitro* screening of relevant drugs during anesthesia and surgery, no pharmacological agent reduced CD11b expression in a relevant manner (**Table 2**). By contrast, protamine and the combination of protamine and heparin increased CD11b expression (**Table 2**).

**Table 2:** Analysis of the effect of various pharmacological agents relevant to perioperative management on neutrophil activation and formation of platelet-neutrophil complexes (PNCs).

	CD11b		CD61	
Cefazolin	0.13	± 0.13	−0.04	± 0.29
Etomidate	0.04	± 0.06	−0.16	± 0.09
Fentanyl	0.01	± 0.05	−0.03	± 0.15
Gelafundin	0.20	± 0.09	0.04	± 0.22
Heparin 1	0.02	± 0.02	−0.10	± 0.09
Heparin 30	−0.01	± 0.05	−0.17	± 0.04
Heparin 100	−0.05	± 0.05	−0.39	± 0.09
Inzolen	0.12	± 0.05	1.04	± 0.39
Jonosteril	0.15	± 0.04	0.10	± 0.23
Noradrenaline	−0.05	± 0.08	−0.20	± 0.19
Pancuronium	0.05	± 0.04	0.26	± 0.24
Priming solution	0.15	± 0.06	0.11	± 0.14
Propofol	0.02	± 0.07	−0.22	± 0.08
Protamine	0.39	± 0.15	0.16	± 0.48
Protamine + Heparin	0.37	± 0.17	−0.30	± 0.31
Remifentanil	−0.03	± 0.07	−0.09	± 0.23
Sevoflurane	0.05	± 0.04	−0.06	± 0.07
Tranexamic acid	0.05	± 0.08	0.04	± 0.14

Blood from healthy volunteers was precincubated with the listed substances and subsequently stimulated with 1 µM platelet-activating factor (PAF). Data is normalized, 0.00 equals the respective PAF-stimulated sample without a pharmacological inhibitor except the corresponding buffer controls, blue indicates a decrease, green indicates an increase. For CD11b, the median fluorescence intensity has been analyzed, for CD61, the percentage of PNCs is reported. Mean ± SD, n = 4.

**Figure 3:**
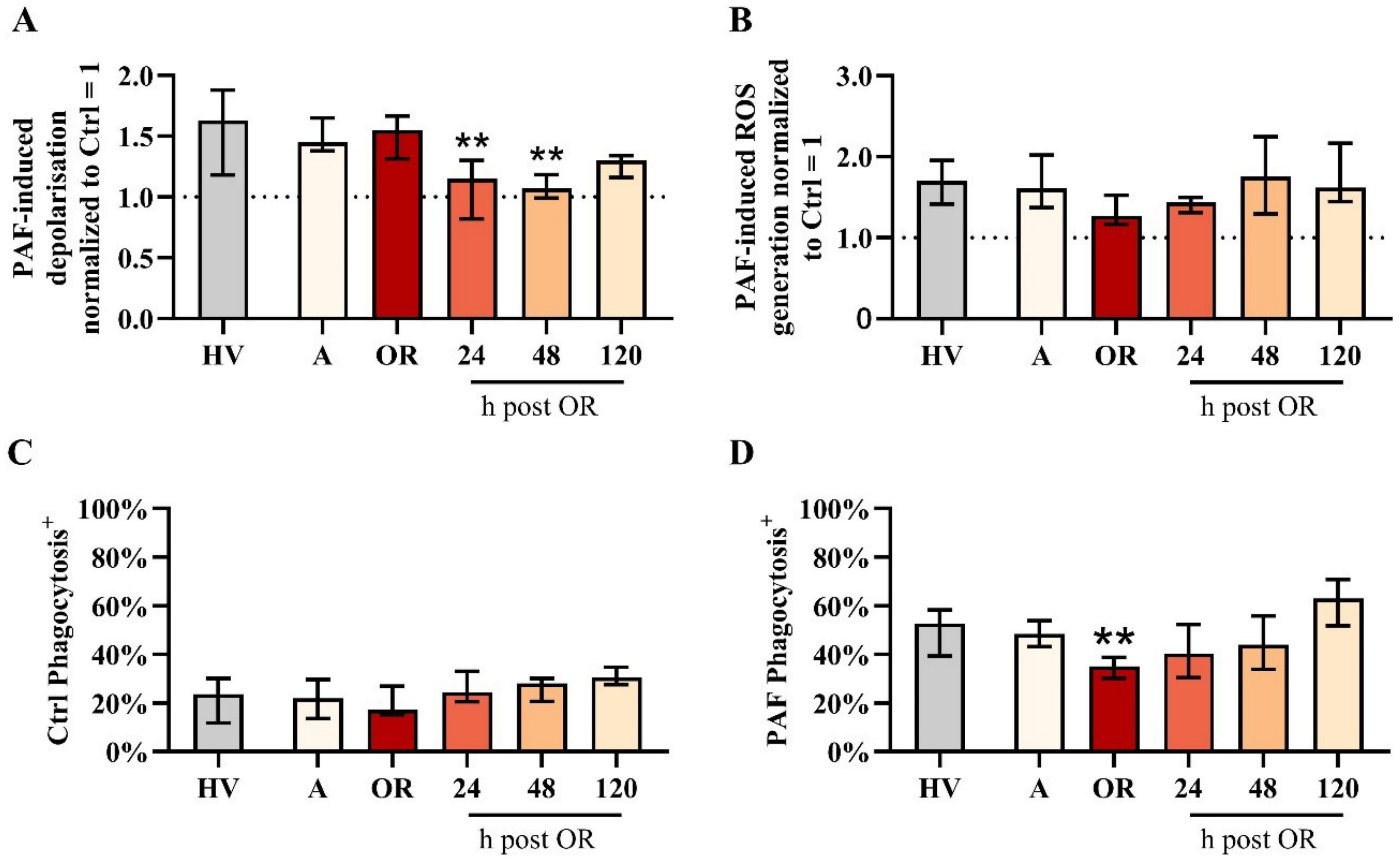
Analysis of neutrophil properties in patients with cardiac surgery on admission (A), 45 min after the initiation of extracorporeal circulation in the operation room (OR), and 24, 48, and 120 h after the end of surgery compared to healthy volunteers (HVs). **(A)** PAF-induced depolarization, **(B)** PAF-induced generation of reactive oxygen species (ROS), **(C)** phagocytic activity in samples exposed to buffer control (ctrl), and **(D)** phagocytic activity in blood exposed to 1 µM platelet-activating factor (PAF). Median with interquartile range, n = 14. HV vs. A: unpaired t test; A vs. OR, 24, 48, and 120 h post OR: ordinary one-way ANOVA with ** indicating a p < 0.01.

Subsequently, changes in neutrophil physiology with respect to the PAF-induced responses of the MP and pH_i_ were assessed. In this regard, PAF-induced depolarization remained stable during surgery but was severely impaired on the first two postoperative days (**Figure 3**). By contrast, neither baseline levels nor PAF-induced changes of pH_i_ and cellular shape were recorded perioperatively (**Supplementary Figures 1**, **2**).

Neutrophil function regarding PAF-induced ROS generation remained stable despite a small but nonsignificant reduction during surgery (**Figure 3**). Phagocytic activity in resting cells was unaltered perioperatively. However, PAF-stimulated neutrophils exhibited reduced phagocytic activity intraoperatively (**Figure 3**).

### 3.3 Perioperative PNC formation

**Figure 4:**
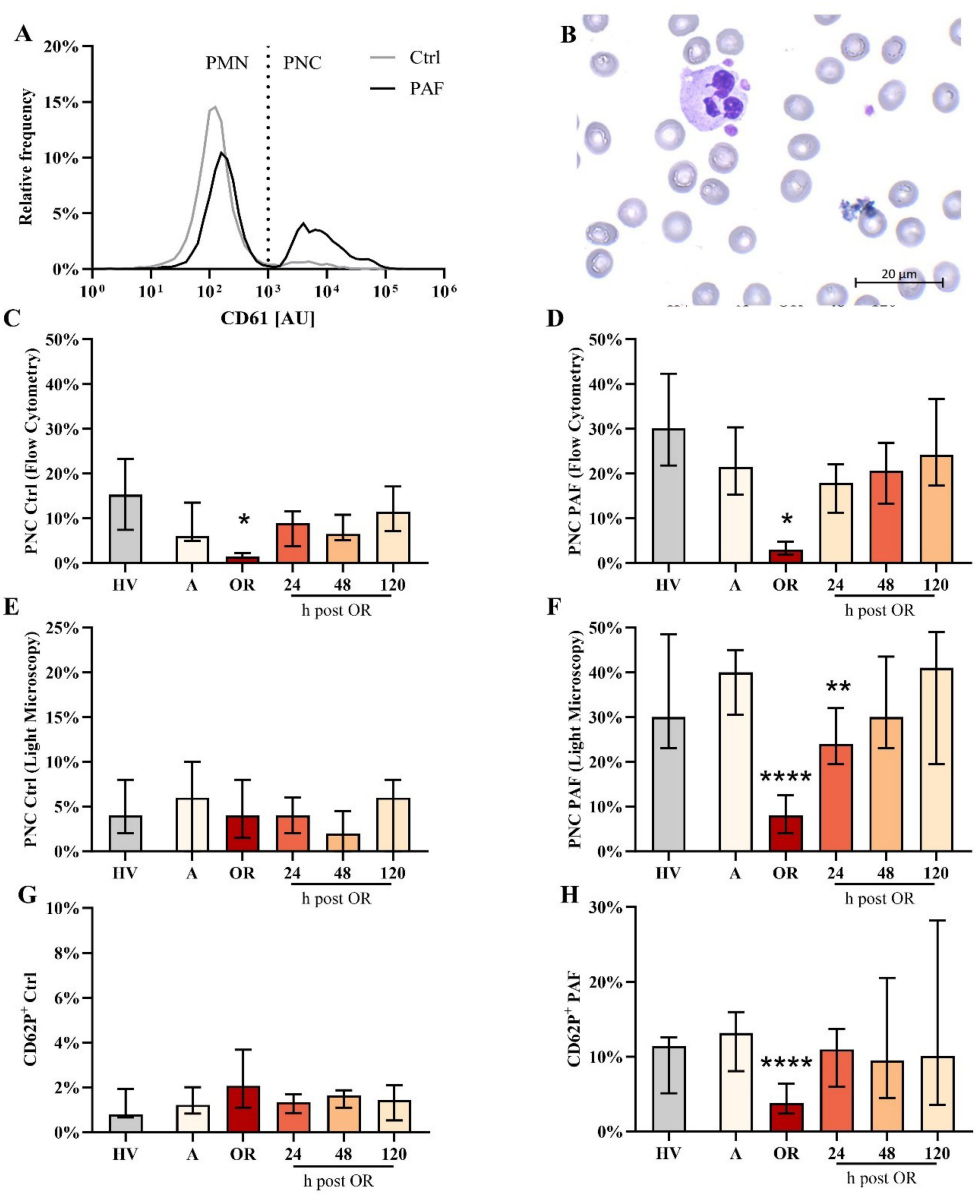
Analysis of platelet-neutrophil complex (PNC) formation and platelet activation in blood from patients with cardiac surgery on admission (A), 45 min after the initiation of extracorporeal circulation in the operation room (OR), and 24, 48, and 120 h after the end of surgery compared to healthy volunteers (HV). **(A)** Representative distribution of CD61 as a platelet-lineage marker on neutrophils from a HV in blood exposed to buffer control (ctrl) or 1 µM platelet-activating factor (PAF). **(B)** Representative PNC as detected by light microscopy. Flow cytometric analysis of PNC formation in samples stimulated with **(C)** Ctrl or **(D)** PAF. Confirmation of PNC formation by light microscopy in samples stimulated with **(E)** Ctrl or **(F)** PAF. Analysis of platelet activation indicated by CD62P expression in platelets exposed to **(G)** buffer control or **(H)** PAF. Median with interquartile range, n = 14. HV vs. A: unpaired t test; A vs. OR, 24, 48, and 120 hours post OR: ordinary one-way ANOVA with *, **, or **** indicating a p value of < 0.05, <0.01, and <0.0001, respectively. AU, arbitrary units.

Surprisingly, PNC formation was strongly impaired intraoperatively but not pre- or postoperatively (**Figure 4**). This phenomenon could be confirmed by two independent techniques (flow cytometry and conventional light microscopy). Moreover, PNC formation was also massively restricted despite the presence of PAF (**Figure 4**). In accordance, the PAF-elicited increase in CD62P-positive platelets was significantly diminished intraoperatively (**Figure 4**). Of note, in PNCs induced by PAF, the number of platelets attached to neutrophils did not differ significantly intraoperatively (data not shown). PNC formation was diminished by ropivacaine, iloprost, and an antibody against CD62P (**Table 1**). In blood from HVs that was stimulated with PAF, the addition of drugs relevant to anesthesia and surgery did not reduce PNC formation in a relevant manner, despite the addition of high doses of heparin (**Table 2**).

**Figure 5:**
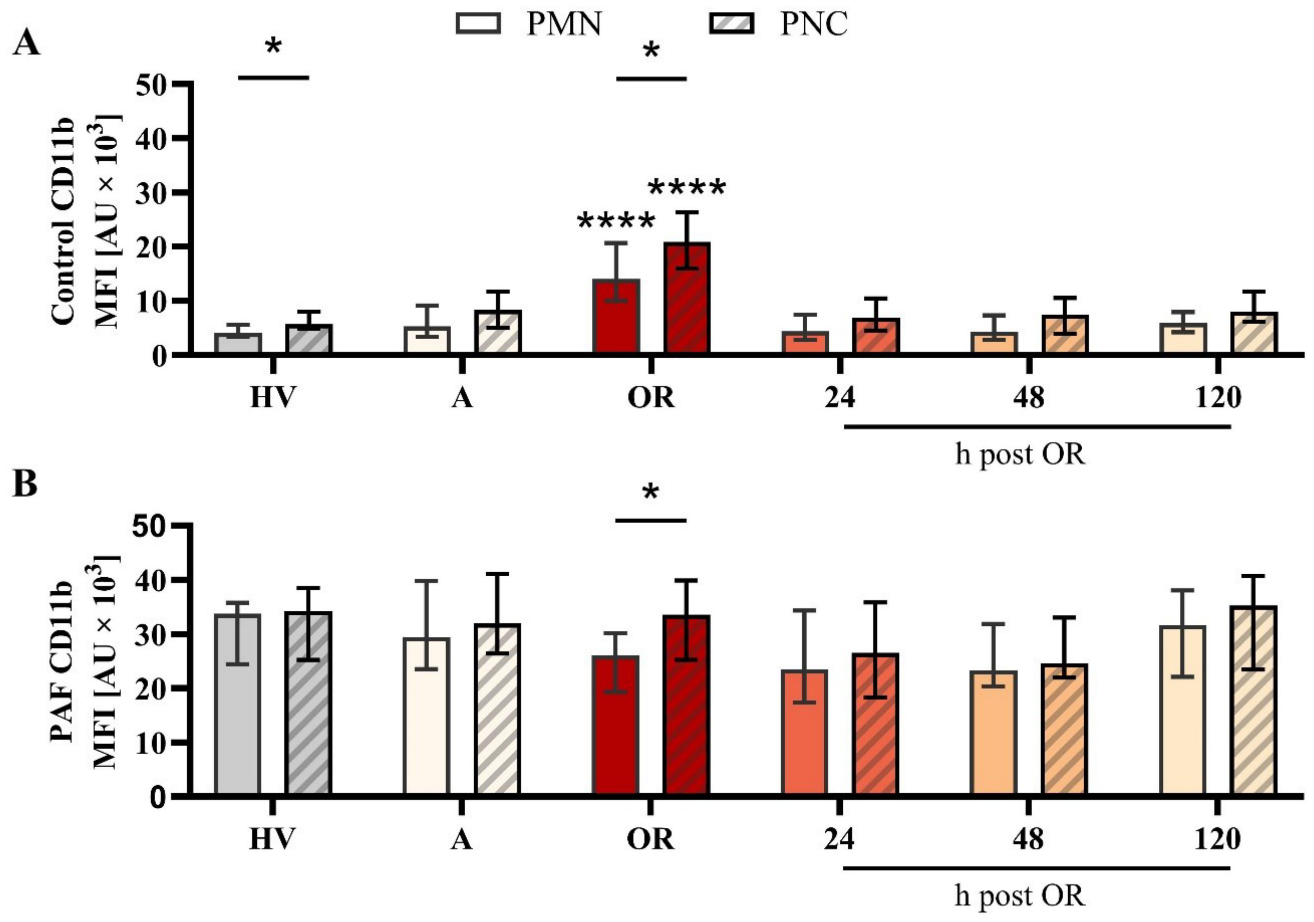
Analysis of CD11b expression on neutrophils without (PMN) or with platelet-neutrophil complex (PNC) formation in samples from patients with cardiac surgery on admission (A), 45 min after the initiation of extracorporeal circulation in the operation room (OR), and 24, 48, and 120 h after the end of surgery compared to healthy volunteers (HV). Blood samples were stimulated with **(A)** buffer control or **(B)** 1 µM platelet-activating factor (PAF). Frequency of PMN and PNCs are shown in **Figure 4** C) + D) PMN vs. PNC: unpaired t test; HV vs. A: unpaired t test; A vs. OR, 24, 48, and 120 h post OR: ordinary one-way ANOVA. * and **** indicating a p value of < 0.05 and <0.0001, respectively. MFI, median fluorescence intensity.

**Figure 6:**
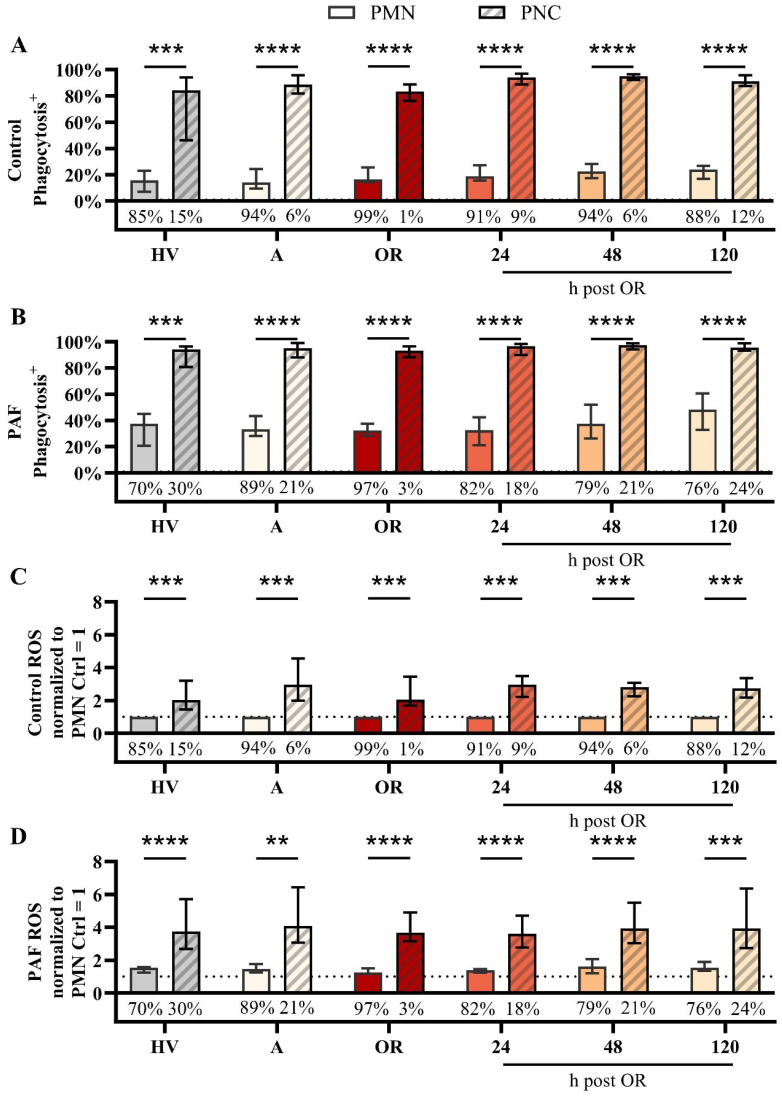
Analysis of neutrophils without (PMN) or with platelet-neutrophil complex (PNC) formation in samples from patients with cardiac surgery on admission (A), 45 min after the initiation of extracorporeal circulation in the operation room (OR), and 24, 48, and 120 h after the end of surgery compared to healthy volunteers (HV). Phagocytic activity in samples exposed to **(A)** buffer control or **(B)** 1 µM platelet-activating factor (PAF). Generation of reactive oxygen species (ROS) by neutrophils exposed to **(C)** buffer control or **(D)** 1 µM PAF. Percentage values below the bar chart indicate the frequency of PMN and PNCs as shown in **Figure 4** C) + D) PMN vs. PNC: A) + B) + D) unpaired t test, C) One sample Wilcoxon test with **, ***, and **** indicating a p value of < 0.01, < 0.001, and <0.0001, respectively.

In a more detailed analysis, the differences between PNCs and PMNs (neutrophils without attached platelets) were investigated. With regard to variations in the phenotype, PMN and PNCs did not differ regarding CD10, CD11b, or CD66b, despite an increase in CD11b in PNCs intraoperatively in comparison to PMN (**Figure 5**, **Supplementary Figures 3**, **4**). However, PNCs exhibited a strong augmentation in ROS generation and phagocytic activity before, during, and after cardiac surgery (**Figure 6**).

## 4 Discussion

Cardiac surgery, including extracorporeal circulation with accompanying IRI, results in transient acute systemic inflammation. The findings of acute upregulation of neutrophil activity (e.g., CD11b) with a delayed upregulation of humoral markers of inflammation (e.g., CRP) are in accordance with previous studies (24–27). The present study adds maintained responsiveness of neutrophils to the proinflammatory mediator PAF with regard to CD11b and CD66b, but not CD10. The biology of the last remains to be further elucidated, although, reduced CD10 upregulation upon stimulation has previously been reported in patients with sepsis (28). Of note, the decreased median surface CD10 expression may also be caused by the release of immature neutrophils from the bone marrow (29). One could speculate that this reduced neutrophil response during the first two postoperative days (days three and four were not assessed) might be indicative of a temporary immunological dysfunction. Interestingly, these findings coincided with a decrease in PAF-induced depolarization, a phenomenon that was previously observed in experimental sepsis and hemorrhagic shock (13, 30). However, it should be noted that reduced cellular depolarization did not result in a (significant) reduction of ROS generation. Because a large body of literature suggested that neutrophil depolarization is associated with ROS generation by activation of NADPH oxidase, this issue should be further elucidated (31). Likewise, the biological relevance of the small yet significantly reduced phagocytic activity induced by PAF should be further evaluated and might be associated with decreased PNC generation as discussed below.

PNC formation by PAF was in accordance with previous results (13, 32). Its formation involves the interaction of several receptors and ligands, including PSGL-1 with CD62P (P-selectin), CD40 with CD40L, and TREM1 with TREM1L, among others (33–35). The interaction of (activated) platelets and neutrophils has previously been reported to increase ROS generation and phagocytic activity (35–38), which this study confirmed for PAF stimulation in HVs as well as in patients scheduled for cardiac surgery. In this context, it is of note that in a murine model of myocardial IRI, platelet-derived serotonin induced neutrophil activation, implicating that this platelet-neutrophil interaction might be a potential therapeutic rationale (39). Similarly, targeting platelet-neutrophil interaction has been reported to greatly reduce murine acid-induced acute lung injury (40). Therefore, targeting PNC formation is discussed as a potential target in cardiovascular disease (33). To the best knowledge of the authors, the reduced PNC formation of blood samples with or without exposure to PAF has not previously been reported in the context of cardiac surgery and could not be explained by a large decrease in the platelet or neutrophil count or a drug effect as briefly investigated by an *in vitro* screening. Interestingly, another study reported increased PNC formation after the implementation of left ventricular assist devices and stable PNC formation in patients with coronary artery bypass grafting and/or aortic valve replacement (41, 42). Similarity, a small yet significant increase in platelet-monocyte complex formation has been reported previously (43). The reduction in PNC formation as a key finding of the present study was validated by two different methods (flow cytometry and conventional light microscopy) and should be confirmed as well as investigated with a higher time resolution in terms of both the frequency and a longer duration and with regard to clinical implications such as a temporary immune dysfunction and/or platelet dysfunction.

From the point of view of the authors, the significantly reduced activity of PNC formation with or without additional stimulation is a rather unexpected finding of the study, despite no major change in the cell count. The performed *in vitro* studies could not identify a surgery- or anesthesia-related drug, including heparin, which would have prevented PNC formation. However, because the reduced PNC formation was somewhat surprising, the present study did not include a comprehensive analysis of platelet activity, such as other platelet activation markers besides CD62P. Moreover, short-term *in vitro* exposure to anesthesia-related drugs does not accurately replicate the *in vivo* conditions, thereby limiting the current explanatory power of the present findings with regard to potential drug effects. Follow-up studies need to further elucidate the underlying mechanisms of the reduced platelet-neutrophil interaction, for example, interaction with the extracorporeal circulation, and to investigate their potential benefits (e.g., amelioration of acute neutrophil-driven inflammation) and threats (e.g., increased vulnerability to invading pathogens, increased bleeding complications).

The present study has several strengths and limitations. As a prospective monocentric observational study, the generalizability is limited. Even so, in contrast to patients with severe injuries, including patients with scheduled surgery, including IRI and tissue damage, the patients of the present study themselves can serve as control, thereby serving as an ideal pre-post comparison. Further studies need to confirm these findings in a larger patient group, including sub-group analyses (sex, smokers, etc.) and/or patients with different forms of cardiac surgery. Moreover, larger cohorts are needed to reliable determine whether data in the context of immunomonitoring in patients with cardiac surgery during surgery is distributed parametric, which was assumed in the analysis of the present study. Similarly, the effect of perioperative drug administration has only been investigated in a small cohort without statistical analysis and in healthy volunteers, which limits the meaningfulness of the data set and thus requires follow-up studies. Moreover, the combination of certain drugs in blood samples from patients, also with different doses, should be further investigated. Monitoring neutrophils by flow cytometry has several implications: While the focus on a single method has its limitation, flow cytometry is a widely established method allowing the synchronous assessment of multiple parameters in thousands of cells per second. Of note, the precise multiparametric description of neutrophil-driven inflammation results in the generation of novel biomarkers to evaluate different surgical procedures such as minimal-invasive surgical approaches. However, it should be noted that the casual relationship between innate immune system activation and organ injury remains a matter of debate (44). Moreover, the stimulation of platelets and neutrophils was limited to PAF, which is one important but not the sole inflammatory mediator during systemic inflammation. The present patient group did not suffer from acute infectious-associated complications. However, one could speculate that neutrophil-driven monitoring approaches might aid in the early recognition of infectious complications, which should be investigated in patient groups with a higher risk of such developments (e.g., patients with colorectal surgery, vascular patients with or without diabetes with wound healing problems, burn patients). Last, follow-up studies need to address the underlying mechanistics of the present surprising findings, e.g., the unresponsiveness in changes in MP postoperatively and failure to form PNCs intraoperatively.

## 5 Conclusion

Cardiac surgery and associated IRI triggers prompt neutrophil activation, which precedes the upregulation of traditional markers of inflammation such as CRP, IL6, and PCT. Therefore, monitoring neutrophil activity may serve as a useful biomarker to monitor the inflammatory response postoperatively, and, perhaps, even monitor potential complications such as infections. Intraoperatively, the interaction of platelets and neutrophils is altered, which might affect neutrophil effector function. Further studies need to elucidate the mechanism and investigate the possibility to modulate platelet-neutrophil interaction as a potential treatment rationale to modulate excessive inflammation in the perioperative context.

## Data Availability

The original contributions presented in the study are included in the article/**Supplementary Material**. Further inquiries can be directed to the corresponding author.
