# Supplementary material for: Multimodal monitoring of neutrophil activity during cardiac surgery
Source: Front Immunol. 2025 Mar 13;16:1504944. doi: 10.3389/fimmu.2025.1504944 (PMC11947689; doi:10.3389/fimmu.2025.1504944)
Supplement: Supplementary file 2 [file SupplementaryFile1.pdf]

Supplementary Material

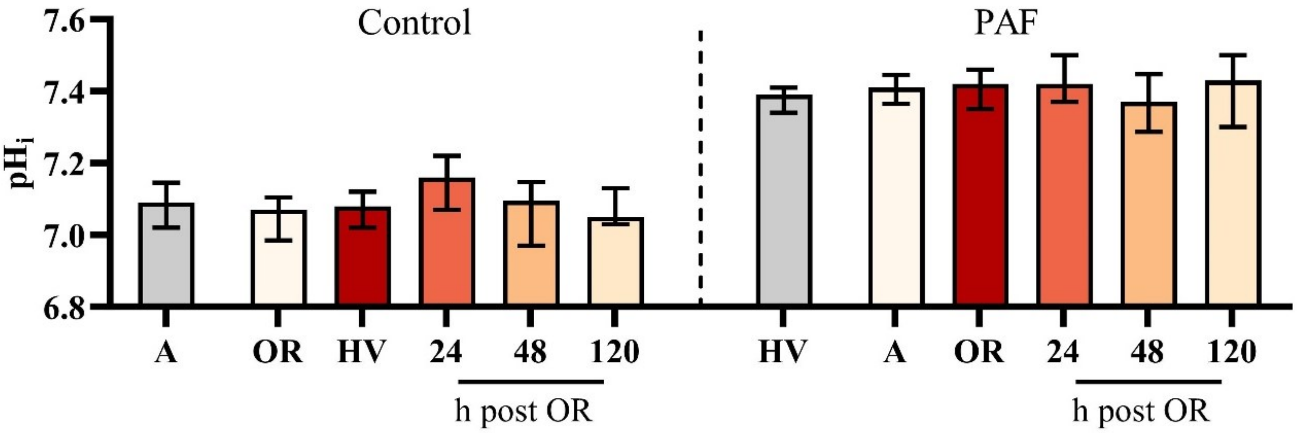

Supplemental Figure 1: Analysis of the neutrophil intracellular pH (pH_i) from patients with cardiac surgery on admission (A), 45 min after the initiation of extracorporeal circulation in the operation room (OR), and 24, 48, and 120 h after the end of surgery compared to healthy volunteers (HV). Blood samples were stimulated with buffer control (control, left) or 1 µM platelet-activating factor (PAF, right). Median with interquartile range, n = 14. HV vs. A: unpaired t test; A vs. OR, 24, 48, and 120 h post OR: ordinary one-way ANOVA.

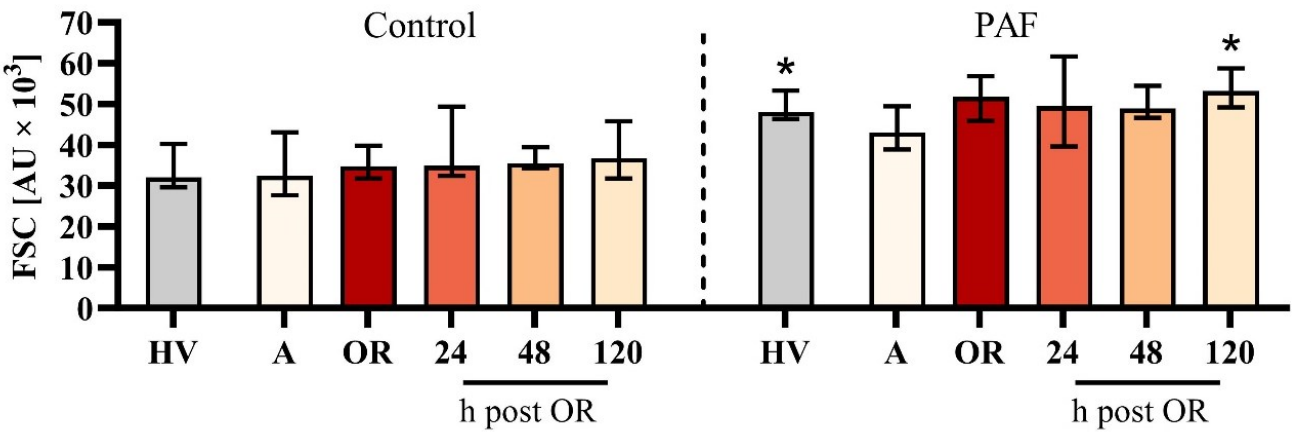

Supplemental Figure 2: Analysis of the neutrophil forward scatter (FSC, as a brief indication of cell shape changes) from patients with cardiac surgery on admission (A), 45 min after the initiation of extracorporeal circulation in the operation room (OR), and 24, 48, and 120 h after the end of surgery compared to healthy volunteers (HV). Blood samples were stimulated with buffer control (control, left) or 1 μM platelet-activating factor (PAF, right). Median with interquartile range, n = 14. HV vs. A: unpaired t test; A vs. OR, 24, 48, and 120 h post OR: ordinary one-way ANOVA. * indicates $p < 0.05$.

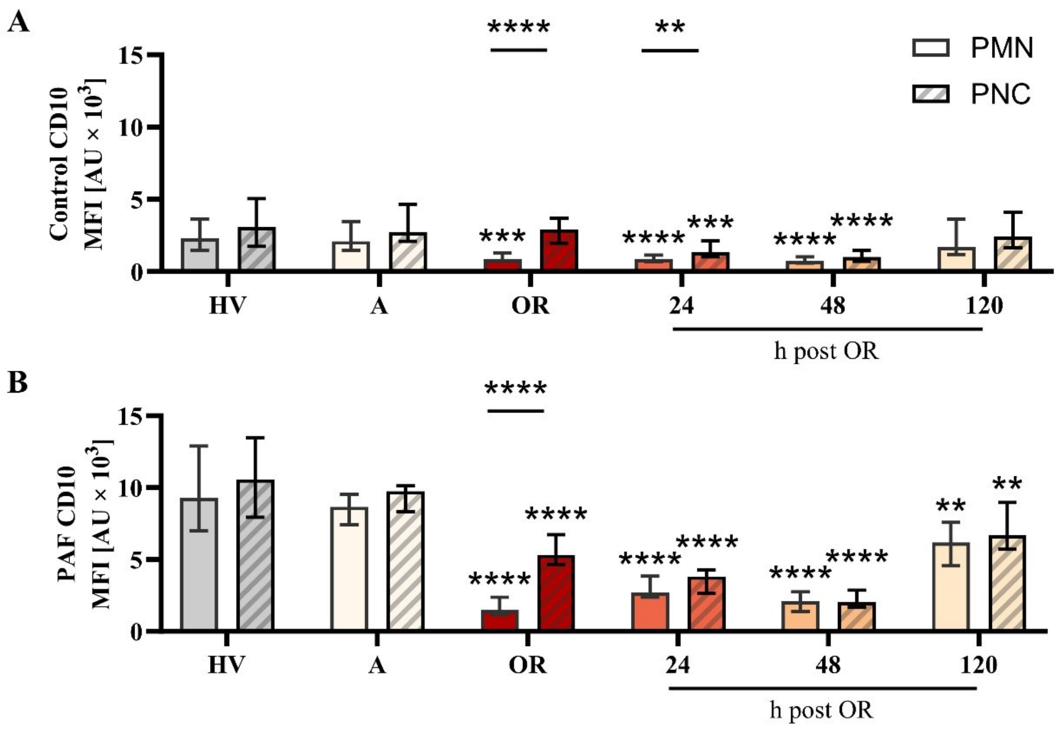

Supplemental Figure 3: Analysis of CD10 expression on neutrophils without (PMN) or with platelet-neutrophil complex (PNC) formation in samples from patients with cardiac surgery on admission (A), 45 min after the initiation of extracorporeal circulation in the operation room (OR), and 24, 48, and 120 h after the end of surgery compared to healthy volunteers (HV). Blood samples were stimulated with A) buffer control or B) 1 µM platelet-activating factor (PAF). PMN vs PNC: unpaired t test; HV vs. A: unpaired t test; A vs. OR, 24, 48, and 120 h post OR: ordinary one-way ANOVA. **, ***, and **** indicating a p value of < 0.01, < 0.001, and <0.0001, respectively.

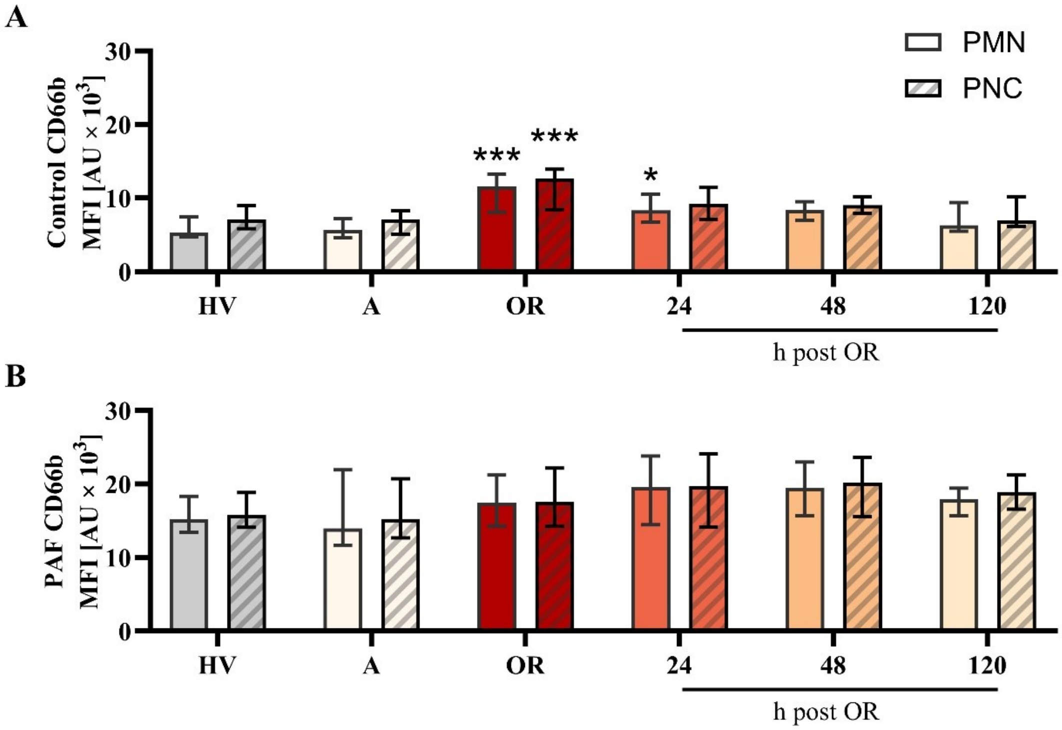

Supplemental Figure 4: Analysis of CD66b expression on neutrophils without (PMN) or with platelet-neutrophil complex (PNC) formation in samples from patients with cardiac surgery on admission (A), 45 min after the initiation of extracorporeal circulation in the operation room (OR), and 24, 48, and 120 h after the end of surgery compared to healthy volunteers (HV). Blood samples were stimulated with A) buffer control or B) 1 µM platelet-activating factor (PAF). PMN vs PNC: unpaired t test; HV vs. A: unpaired t test; A vs. OR, 24, 48, and 120 h post OR: ordinary one-way ANOVA. * and *** indicating a p value of < 0.05 and <0.001, respectively.

Parameter	HV	A	OR	24 h	48 h	120 h
**Leukocytes**	5.6**	7.3	8.4	10.2**	11.9****	7.8
**[Giga/l]**	(5.0|6.3)	(6.3|8.4)	(6.9|10.2)	(8.8|12.8)	(10.5|13.0)	(7.1|9.0)
**Erythro-cytes**	4.6*	5.0	3.4****	3.5****	3.4****	3.4****
**[Tera/l]**	(4.0|5.1)	(4.7|5.6)	(3.0|3.6)	(3.3|3.8)	(3.2|3.7)	(3.1|4.1)
**Hemo-globin**	13.7*	15.7	10.3****	10.8****	10.4****	10.6****
**[g/dl]**	(12.4|16.0)	(14.5|16.9)	(9.0|10.9)	(9.8|11.4)	(9.4|11.1)	(9.7|12.0)
**Hematocrit**	0.4*	0.5	0.3****	0.3****	0.3****	0.3****
**[l/l]**	(0.4|0.5)	(0.4|0.5)	(0.3|0.3)	(0.3|0.3)	(0.3|0.3)	(0.3|0.4)
**MCV**	91.3	88.1	89.2	87.9	89.1	90.3
**[fl]**	(87.7|92.2)	(84.8|90.5)	(85.7|91.1)	(87.3|92.0)	(87.0|94.0)	(86.2|92.2)
**MCH**	30.7	29.9	30.1	30.2	30.2	29.7
**[pg]**	(30.3|31.5)	(29.2|31.9)	(29.3|31.6)	(29.1|31.4)	(29.2|31.9)	(28.9|31.3)
**MCHC**	34.3	34.2	34.0	33.4	33.6	33.6
**[g/dl]**	(33.6|35.1)	(33.5|35.4)	(33.2|35.0)	(33.1|35.0)	(33.3|34.5)	(33.2|34.6)
**Thrombo-cytes**	232.0	225.0	134.5****	140.5****	127.0****	188.0
**[Giga/l]**	(185.0|256.5)	(198.0|259.3)	(120.3|156.3)	(94.8|168.3)	(97.3|150.0)	(116.5|261.0)
**MTV**	10.8	10.6	11.2	11.3	11.5	10.8
**[fl]**	(10.0|11.5)	(10.1|11.2)	(10.5|11.8)	(10.9|11.7)	(10.8|11.9)	(10.1|11.6)
**Lympho-cytes**	27.8	21.6	17.2*	9.7****	11.0****	18.2*
**[%]**	(24.7|31.1)	(17.8|29.7)	(14.0|20.8)	(5.7|11.5)	(8.5|15.0)	(14.7|20.3)
**Neutro-phils**	57.4	67.7	77.9	80.9**	78.2	64.3
**[%]**	(56.3|63.3)	(56.0|70.8)	(69.9|81.0)	(76.6|86.4)	(73.8|80.2)	(61.5|69.2)
**Basophils**	0.7	0.7	0.5**	0.3****	0.3***	0.9
**[%]**	(0.6|1.1)	(0.6|1.1)	(0.3|0.7)	(0.1|0.4)	(0.3|0.5)	(0.5|1.2)
**Eosinophils**	2.7	1.4	1.0	0.1**	0.3	3.1
**[%]**	(1.4|4.5)	(1.1|3.9)	(0.7|1.4)	(0.0|0.2)	(0.3|1.9)	(1.9|4.4)

**Monocytes**	9.1	8.9	3.2****	9.4	10.8	11.8*
**[%]**	(7.4|10.4)	(6.3|10.2)	(2.1|4.4)	(7.9|13.2)	(6.8|12.7)	(10.1|13.1)

Supplemental Table 1: Complete blood count analyzing patients with cardiac surgery on admission (A), 45 min after the initiation of extracorporeal circulation in the operation room (OR), and 24, 48, and 120 h after the end of surgery in comparison to age- and sex-matched healthy volunteers (HV). Median with interquartile range, n = 14. HV vs. A: unpaired t test; A vs. OR, 24, 48, and 120 h post OR: ordinary one-way ANOVA in conjunction with a Dunnett's multiple comparison test. MCV = mean corpuscular volume, MCH = mean corpuscular hemoglobin, MCHC = mean corpuscular hemoglobin concentration, and MTV = mean thrombocyte volume.

Parameter	HV	A	OR	24 h	48 h	120 h
**Urea**		5.6	8.2	6.8	7.7	6.0
**[mmol/l]**		(4.9|8.5)	(4.2|9.5)	(4.7|11.4)	(6.0|13.2)	(5.6|9.0)
**Creatinine**	79.8	75.0	71.5	79.0	76.0	76.0
**[µmol/l]**	(66.2|90.5)	(65.8|96.0)	(63.8|86.5)	(62.8|100.3)	(59.3|97.0)	(68.0|92.5)
**ALT**		23.0	25.0	22.0	20.0	39.0
**[U/l]**		(12.8|32.0)	(16.0|35.5)	(16.0|36.5)	(16.0|28.0)	(34.0|70.0)
**CK**		103.0	388.0*	786.5****	713.5****	84.0
**[U/l]**		(78.5|146.5)	(351.0|705.0)	(533.5|1.098.0)	(367.3|921.8)	(68.0|143.0)
**CK-MB**		2.0	19.6****	17.0****	8.2	1.8
**[U/l]**		(1.4|3.6)	(13.9|31.6)	(12.3|22.5)	(5.0|11.3)	(1.2|2.5)
**Troponin-T**		10.5	409.0	454.5**	374.0	293.0
**[ng/l]**		(7.0|19.0)	(238.5|1.054.0)	(291.0|740.5)	(207.8|644.5)	(156.5|507.0)
**aPTT**		29.4	40.1*	37.8	45.0**	42.3**
**[sec.]**		(27.7|31.3)	(35.4|59.6)	(32.4|44.1)	(38.6|52.3)	(28.4|56.9)
**INR**		1.0	1.3	1.1	1.1	1.1
		(1.0|1.0)	(1.3|1.3)	(1.1|1.2)	(1.1|1.2)	(1.0|1.4)
**Sodium**	141.0*	139.0	137.5	140.0	137.0	138.0
**[mmol/l]**	(139.0|142.0)	(138.0|140.5)	(131.0|139.5)	(138.0|144.0)	(133.8|140.3)	(136.8|140.0)
**Potassium**	4.1	4.0	5.0***	4.7***	4.5**	4.1
**[mmol/l]**	(4.0|4.3)	(3.9|4.2)	(4.8|5.2)	(4.3|4.8)	(4.2|4.9)	(3.7|4.2)
**Calcium**	1.25	1.23	1.17**	1.15****	1.15****	1.19
**[mmol/l]**	(1.22|1.28)	(1.19|1.25)	(1.09|1.20)	(1.11|1.20)	(1.12|1.17)	(1.18|1.22)
**Glucose**	104.0	118.5	124.0	152.5*	168.5**	158.0
**[mg/dl]**	(90.8|124.3)	(98.8|141.3)	(122.0|176,0)	(137.3|195.3)	(142.5|197.0)	(125.0|201.5)

Supplemental Table 2: Organ function parameters, electrolytes, and glucose analyses of patients with cardiac surgery on admission (A), 45 min after the initiation of extracorporeal circulation in the operation room (OR), and 24, 48, and 120 h after the end of surgery in comparison to age- and sex-matched healthy volunteers (HV). Median with interquartile range, n = 14. ALT = alanine transaminase, CK = creatine kinase, CK-MB = creatine kinase-MB, aPTT = activated partial thromboplastin time, INR = international normalized ratio. HV vs. A: unpaired t test; A vs. OR, 24, 48, and 120 hours post OR: ordinary one-way ANOVA in conjunction with a Dunnett’s multiple

comparison test with *, **, ***, and **** indicating a p value of < 0.05, < 0.01, < 0.001, and <0.0001, respectively.

	CD10 A	CD10 OR	CD11b A	CD11b OR	PNC A	PNC OR
CRP 24 h	0.47	−0.27	0.69**	0.52	−0.25	0.72**
CRP 48 h	0.33	0.11	0.42	0.23	−0.28	0.62
CRP 120 h	−0.08	0.31	−0.21	0.12	0.4	−0.12
IL6 24 h	0.39	0.51	0.23	0.18	0.09	0.27
IL6 48 h	−0.24	0.19	−0.48	−0.23	0.60*	−0.09
IL6 120 h	0.36	0.27	0.12	0.32	0.46	0.15
CK 24 h	−0.06	−0.19	−0.28	−0.18	0.28	0.08
CK 48 h	−0.01	0.13	−0.31	−0.34	0.45	−0.08
CK 120 h	0.37	−0.11	0.24	−0.05	0.13	0.69**
MMP9 24 h	−0.27	0.70*	−0.39	−0.26	0.05	−0.46
MMP9 48 h	0.02	0.02	0.1	0.43	0.09	−0.14
MMP9 120 h	0.44	0.3	0.32	−0.1	−0.24	0.15
OR time	−0.23	0.28	−0.39	−0.43	−0.28	−0.10
LOS ICU	0.06	0.54*	−0.17	−0.03	0.48	−0.05

Supplemental Table 3: Correlation analysis for representative neutrophil-related biomarkers (expression of CD10 and CD11b on neutrophils and formation of platelet-neutrophil complexes (PNC)) on admission (A), 45 min after the initiation of extracorporeal circulation in the operation room (OR), and 24, 48, and 120 h. Pearson r with * and **indicating a p value of < 0.05 and < 0.01, respectively

Drug	Vendor	Concentration
Cefazolin	Cefazolin HEXAL 2 g, Fresenius Kabi, Bad Homburg, Germany,	1 mM
Etomidate	Etomidat-Lipuro 2 mg/mL, B. Braun Melsungen AG, Melsungen, Germany	10 µM
Fentanyl	FENTANYL 50 µg/mL Injektionslöung Amp, Hameln Pharma, Hameln, Germany	100 nM
Gelofusine	Gelofusine (Gelafundin ISO 40 mg/mL Ecoflac plus Inf) B. Braun Melsungen AG, Melsungen, Germany	4 µL in 40 µL
Heparin	Heparin Natrium Braun 100 000 I.e. Ampullen, B. Braun, Melsungen AG, Deutschland	1, 30, 100 IE/mL
Jonosteril	JONOSTERIL Beutel Infusionslösung, Fresenius Kabi Deutschland GmbH, Bad Homburg, Germany	4 µL in 40 µL
Norepinephrine	Arterenol 1 mg/mL, Cheplapharm Arzneimittel GmbH, Greifswald, Germany	100 nM
Mannitol	Osmofundin 15% N Ecoflac plus 250 mL, Serag-Wiesner, Naila, Germany	15%
Pancuronium	Pancuronium 4 mg/2 mg, Inresa Arzneimittel, Freiburg, Germany	0,2 mM
Priming solution	Priming volume in vivo 1250 mL, Jonosteril 1000 mL, Osmofundin 250 mL, Heparin 5000 IE, Tranexamsäure 1000 mg	4 µL in 40 µL
Propofol	Propofol-Lipuro 10 mg/mL, B. Braun Melsungen AG, Melsungen, Germany	100 µM
Protamine	Protaminsulfat LEO 1400 Heparin-Antidot I.E./ML; Leo Pharma GmbH, Neu-Isenburg, Germany	30 IE/mL blood
Protamine + Heparin	See above	each 30 IE/mL
Remifentanil	Remifentanil HEXAL 2 mg, Hexal AG, Holzkirchen, Germany	500 nM
Sevoflurane	Sevofluran 100%, Baxter, Unterschleißheim, Germany	50 mM
Tranexamic acid	Tranexamsäure 100 mg/mL, Carinopharm, Eime, Germany	1 mM

Supplemental Table 4: Drugs and their respective concentration for the in vitro screening as reported in table 2
